# GRANDPARENTS’ ROLES IN THE CONTEXT OF ADVERSE CHILDHOOD EXPERIENCES: THE VOICES OF EMERGING ADULTS

**DOI:** 10.1093/geroni/igz038.2342

**Published:** 2019-11-08

**Authors:** Athena Chung Yin Chan, Doris Leung, Bessie Chan, Grace Wing Ka Ho

**Affiliations:** 1 Department of Family Social Science, University of Minnesota, Saint Paul, Minnesota, United States; 2 School of Nursing, The Hong Kong Polytechnic University, Kowloon, Hong Kong

## Abstract

The influence of grandparents, in the context of adverse childhood experiences (ACEs), is largely understudied. With strong kinship in Asian families, grandparents may provide a crucial resource to their grandchildren; not limited to those living together, but having close emotional proximity. This qualitative study used secondary analysis to explore the roles of grandparents, upon reflection of participants’ childhood adversities. Semi-structured interviews were conducted with 19 emerging adults, between 18 and 24 years old in Hong Kong, China. Participants were eligible if they: (1) reported at least one ACE, namely, abuse (physical, emotional, sexual), neglect (physical, emotional), witnessing domestic violence, or a dysfunctional household (due to parental divorce/death, household member substance use, incarceration, mental illness); and (2) described their interactions with grandparents during the interviews. Participants were asked to think of a challenging time during their childhood, and strategies they used to overcome them. All interviews were audio-recorded and transcribed verbatim. Data regarding the interactions with grandparents were coded and analyzed by four researchers following interpretive description. Preliminary findings described four primary roles grandparents played in the context of ACEs, which were sometimes positive and/or negative. Grandparents were portrayed as being unique persons in participants’ lives that influenced how they faced their childhood adversities. We will discuss how grandparents’ stewardship may significantly shape cultural patterns of how families cope with ACEs. In particular, our findings, examined against literature, will discuss how grandparents may enhance resilience of young people with ACEs.

